# COPING BEHAVIORS THAT INCREASE STRESS AMONG OLDER ADULTS

**DOI:** 10.1093/geroni/igz038.3475

**Published:** 2019-11-08

**Authors:** G Rainville, Alicia R Williams, Lona Choi-Allum

**Affiliations:** AARP, Washington, District of Columbia, United States

## Abstract

This study examined the efficacy of a series of 28 behaviors (e.g., comfort eating, attending worship services, getting a massage, etc.) in moderating the perception of stress among older adults. First, 28 individual behaviors were assessed to determine whether they buffered or exacerbated the impact of an objective stress measure (i.e., the count of stressful life events) on perceptions of stress (measured using Cohen’s Perceived Stress Scale [PSS]). A full sample analysis used data from 1,000 randomly selected U.S. adults age 40 and older, but subsequent analyses explored coping behaviors for two age groups—those age 40 to 59 and those 60 and older. In the full sample analysis, multiple moderating conditions were noted including stress-buffering for worship service attendance, recreational shopping, and getting a massage. Also among the full 40+ sample, stress-exacerbation was noted for social media use and coping by “overreacting to things.” Factor analysis (employing a polychoric correlation matrix) reduced the 28 individual behaviors into 9 clusters comprised of related behaviors and representing a general coping approach. Looking within the age groups, significant stress-buffering was limited to those age 60 and older for two coping approaches—a “Self-Care and Travel” approach and an Inspirational approach (e.g., praying, attending church, etc.). For both age subgroups there was no coping approach, not even the hedonistic “blowing off steam” approach, that was found to exacerbate the impact of stressful life events on the perception of stress.

